# Polymeric Films Loaded with Vitamin E and *Aloe vera* for Topical Application in the Treatment of Burn Wounds

**DOI:** 10.1155/2014/641590

**Published:** 2014-01-12

**Authors:** Gabriela Garrastazu Pereira, Sílvia Stanisçuaki Guterres, Anna Giulia Balducci, Paolo Colombo, Fabio Sonvico

**Affiliations:** ^1^Faculdade de Farmácia, Universidade Federal do Rio Grande do Sul, Avenida Ipiranga, 2752, 90610-000 Porto Alegre, RS, Brazil; ^2^Department of Pharmacy, University of Parma, Via Università, 12, 43121 Parma, Italy; ^3^Graduate School of Health-Pharmacy, University of Technology Sydney, 15 Broadway, Ultimo, NSW 2007, Australia

## Abstract

Burns are serious traumas related to skin damage, causing extreme pain and possibly death. Natural drugs such as *Aloe vera* and vitamin E have been demonstrated to be beneficial in formulations for wound healing. The aim of this work is to develop and evaluate polymeric films containing *Aloe vera* and vitamin E to treat wounds caused by burns. Polymeric films containing different quantities of sodium alginate and polyvinyl alcohol (PVA) were characterized for their mechanical properties and drug release. The polymeric films, which were produced, were thin, flexible, resistant, and suitable for application on damaged skin, such as in burn wounds. Around 30% of vitamin E acetate was released from the polymeric films within 12 hours. The *in vivo* experiments with tape stripping indicated an effective accumulation in the *stratum corneum* when compared to a commercial cream containing the same quantity of vitamin E acetate. Vitamin E acetate was found in higher quantities in the deep layers of the *stratum corneum* when the film formulation was applied. The results obtained show that the bioadhesive films containing vitamin E acetate and *Aloe vera* could be an innovative therapeutic system for the treatment of burns.

## 1. Introduction

Burns are among the most complex and harmful physical injuries to clinically evaluate and manage. In addition to pain and distress, a large burned area will leave the patient with visible physical scars and invisible psychological sequelae [1, 2]. Concerning skin damage, the treatment of burns is complex and painful and requires the use of several drugs administered separately or combined [3].

In human beings, the process of tissue reconstruction is completed with remarkable success, but it is unlikely that the body has exactly the same “tools” (raw materials and environment) to work as before, so there are limits regarding the quality of replication. The dressings are a form of treatment of skin wounds aimed at favoring and supporting the healing process.

Potential vectors for the controlled release of substances for the treatment of skin damage occurring in wounds and burns are polymeric films. Thin films have been extensively used around the world for tissue repair and closure of wounds [3–5]. Polymeric film offers advantages over other pharmaceutical forms, such as liquid or semisolid drug delivery products, as it provides a large surface area of application, adhesion to the damaged tissue, and absorption of exudates [6]. The use of natural polymers is supported by their many desirable properties, such as biocompatibility [7], low irritancy, and lack of toxicity [8], as in the case of polysaccharides. In addition, wound dressings are in some cases able to prevent loss of body fluid [3], prevent exudates build up [9], protect the wounds from external contamination [10], provide sufficient bactericidal effects to inhibit infection [11], and prepare an optimum wound bed for autographing [12, 13].

To reduce pain and accelerate the healing process, many natural substances have been traditionally used and more recently have been scientifically studied, such as *Aloe* [14]. *Aloe vera* has been used in a host of curative purposes including treatment of skin disorders and healing of wounds. The colourless gel that comes from the leaf parenchyma has been used to treat burns because, besides being a potent moisturizing agent, it helps in the healing process of skin lesions and alleviates pain [15–17].

Another natural compound investigated in wound healing is vitamin E, a family of essential micronutrients with strong antioxidant activity composed of lipid-soluble tocopherols and tocotrienols. Vitamin E may assist in wound healing through direct effects on tissue repair and regeneration [18, 19].

In relation to this, the objective of the present work has been to develop and characterize a polymeric film containing *Aloe vera* and vitamin E acetate with the aim of providing an innovative system for burn wound treatment. The two polysaccharides selected to produce the film were hyaluronic acid and sodium alginate. Hyaluronic acid is an extracellular matrix component that forms a pericellular coat on the surface of cells and has been shown to contribute to the skin healing process [9, 20, 21]. Sodium alginate, widely used in food and pharmaceutical industries, has been used in a number of wound treatments, for both acute or chronic wounds, because when it comes into contact with the exudate or blood it forms a protective fibrous gel, which is hydrophilic, hemostatic, and rich in calcium [22]. Polyvinyl alcohol was used as film forming agent and has been often used in combination with other polymers for wound healing applications [23, 24].

## 2. Material and Methods

### 2.1. Materials

*Aloe vera* spray dried powder (200 : 1, aloe : mannitol) was obtained from Brasquim (Porto Alegre, Brazil). Vitamin E acetate (alpha tocopherol acetate) was purchased from ACEF (Fiorenzuola, Italy), hyaluronic acid solution with 1% density 0.900–1.100 g/cm^3^ was obtained from DEG (São Paulo, Brazil); polyvinyl alcohol (PVA) with molecular weight of 83,400 Da was obtained from Nippon Gohsei (Osaka, Japan), sorbitol solution 70% was obtained from ACEF (Fiorenzuola, Italy), alginic acid (Satalgine) was obtained from from Cargill (Saint-Germain-en-Laye, France) and poly(ethylene oxide) water soluble resin (PEO 12 NF, 1000 kDa) was obtained from Union Carbide (Milan, Italy). MilliQ ultrapure water (Millipore, Billerica, USA) was used for all experiments. All other chemicals were of analytical grade.

### 2.2. *Aloe vera* and Vitamin E Acetate Loaded Films Preparation

The composition of the dried films is reported in Table 1. The films were prepared starting from two solutions. 

*Solution A.* Alginate powder was added to 20 mL of 1% w/w solution of hyaluronate and stirred until complete dissolution. *Aloe vera* and vitamin E acetate were then added. 

*Solution B*. In 10 mL of water and PEO, PVA and sorbitol were dispersed under gentle heating until complete solubilization. The polymer solution obtained was allowed to stand for 4 h, until all trapped air bubbles were removed.

Solutions A and B, prepared as described, were mixed under magnetic stirring for 4 hours until reaching homogeneity.

The polymeric films were produced by layering the polymeric viscous solution using a variable open casting knife (gap 2 mm, BYK-Gardner GmbH, Geretsried, Germany) on a polyester translucent polyethylene laminate film (Scotchpak 1220 Backing, 3M Italia, Segrate, Italy) and by subsequent drying for 8 h in an oven (55°C).

### 2.3. Physical Characterization of the Film

For each film produced, at least 3 disks of material (15 mm of diameter) were sampled by punching. Each disk was accurately weighed and its thickness was measured (Absolute Digimatic 547–401, Mitutoyo, Milan, Italy, sensitivity 0.001 mm). Residual water content of each formulation was determined using Karl-Fisher titration (TitroMatic KF 1S, Crison, Spain). Samples were analyzed in triplicate.

### 2.4. Vitamin E Acetate Assay

For each formulation, at least 3 disks (15 mm of diameter, surface area 176.7 mm^2^) were sampled. Each disk was accurately weighed and the sample then dissolved in 10 mL of water: ethanol mixture (20 : 80 v/v) under sonication for 2 h. The solution obtained was analyzed by HPLC in order to determine the amount of vitamin E acetate contained in the film. Vitamin E acetate analysis was performed by HPLC using the following experimental conditions: column LiChrospher 100 RP18: 5 *μ*m, 250 mm × 3 mm (Merck, Germany), mobile phase: methanol : isopropanol (50 : 50 v/v), flow rate of 0.7 mL/min, and UV detection at 285 nm. Samples were filtered through 0.45 *μ*m (Ultracel regenerated cellulose, Microcon Filters, Millipore, USA) and were injected (20 *μ*L).

In these conditions, the retention time was about 6.6 min. System suitability was checked according to the USP 24. The detector response was linear from 3 to 51 *μ*g/mL, with a limit of quantification of 0.42 *μ*g/mL, a limit of detection of 0.13 *μ*g/mL, and *r* ^2^ 0,9995.

The loading of vitamin E acetate in polymeric films was expressed as percentage by weight (% w/w) and as mg/cm^2^.

In order to assess the influence of the tape (Scotch 845, 3M, USA) and of the *stratum corneum* on the vitamin E acetate assay, a vitamin recovery experiment was performed. In brief, tape with or without *stratum corneum* was extracted in methanol for 2 hours after adding a known amount of the vitamin E acetate to obtain a concentration of 25 *μ*g/mL. Samples were analyzed with the HPLC method described above and vitamin E recovery was expressed as the percentage of the expected value.

A commercial cream base (Cetaphil Moisturizing Cream, Galderma, USA) was used to prepare a cream containing 3.6% vitamin E acetate by incorporation. This formulation was used in the studies as comparison.

### 2.5. Scanning Electronic Microscopy Analyses

Square film samples of few mm were put on a sample holder with an adhesive tape without metal coating and were examined with a JEOL JSM 6400 (Tokyo, Japan) scanning electron microscope at an intensity of 15 kV using magnifications from 200 to 10000x.

### 2.6. Atomic Force Microscopy Analyses

The films surface morphology was investigated by atomic force microscopy. For AFM observations, the films were placed on a magnetic sample holder using adhesive tape. The films were examined using an XE-100 AFM (Park Systems Inc., Santa Clara, USA). Images were acquired in air in noncontact mode. The spring constant was typically 0.08 N/m and the samples were scanned at constant force with a low scan rate (0.76 Hz) in order to reduce noise and minimize sample damages. Images were obtained with 256 × 256 pixels resolution and image processing (line-wise flatten only) was performed in XEP-Data (Park System, Suwon, Republic of Korea). Height images were displayed as 3D views. At least three regions of 10 × 10 *μ*m were imaged for each film.

### 2.7. Mechanical Evaluation of Films

Films strips (10 × 70 mm) were fixed between the two clamps of an electronic dynamometer (Acquati, Milan, Italy) provided with a 5 kg load cell. The strips were stretched at the rate of 30 mm/min; tensile strength (Ts_*b*_) and elongation at break (*ε*_*b*_) were calculated as shown in (1) and (2), respectively [25, 26]:

(1) $$\begin{array}{c} {\text{Ts}}_{b}=\frac{F_{b}}{\text{C}s}, \end{array}$$

where Ts_*b*_ (N/mm^2^) is the tensile strength, *F*_*b*_ is the breaking force (N), and C*s* is the cross-sectional area of the sample (mm^2^). Consider

(2) $$\begin{array}{c} \varepsilon_{b}=\frac{\Delta L}{\Delta L_{0}}\cdot 100, \end{array}$$

where *ε*_*b*_ is the elongation at break expressed as percentage, Δ*L* is the increase in length at the breaking point (mm), and *L*_0_ is the original length of the film (mm).

### 2.8. *In Vitro* Vitamin E Acetate Release Studies

A release assay of vitamin E acetate from films and commercial cream containing the same quantity of vitamin E acetate was performed using modified Franz permeation cells with a synthetic membrane of regenerated cellulose with 0.45 *μ*m pores (Millipore) to separate the donor and acceptor compartment. The *in vitro* release studies were conducted on vertical diffusion Franz cells, having a receptor compartment with a capacity of about 12.0 mL and a diffusion area of 2.3 cm^2^ [27, 28]. The cellulose membrane was kept in contact with a receptor solution phosphate buffer pH 7.2 with 10% ethanol and 0.5% polysorbate 80 at a temperature of 37°C (±1°C) and constantly stirred with a magnetic bar at 100 rpm. The acceptor medium was selected by determining the saturation concentration of vitamin E acetate in different candidate receptor solutions, selecting the one that provided the highest solubility, that is, 24.4 mg/mL.

For these studies films were used containing approximately 630 *μ*g vitamin E acetate (approximately 2.0 cm^2^ diameter disks) or as reference product, 70 mg of commercial cream containing the same amount of drug. The material under investigation was placed in the donor compartment and hydrated with 500 *μ*L of phosphate buffer pH 7.2. The film was dipped in the buffer in order to allow drug release from both surfaces and the formation of a gel in contact with the membrane.

At preestablished time intervals (each hour up to 12 hours), 1 mL of the medium was removed and replaced by an equal volume of preheated fresh receptor solution. Samples were filtered through 0.45 *μ*m filters (RC, Millipore) before the determination by HPLC of their free vitamin E acetate content according to the method described before. The results refer to the average of five experiments.

### 2.9. Tape Stripping

Five healthy volunteers (2 males and 3 females) participated in the study. All volunteers provided written consent to participate in the study, having understood the design, objectives, and risks of the study. The participants were aged between 26 and 37 years and had no history of skin disease. Each participant was asked to refrain from applying any topical medicaments to their left and right flexor forearms at least 24 h prior to the experiment. The volar forearm of each participant was wiped with water and soap to remove any sebaceous lipids or contaminants on the skin surface. The participant extended both forearms over a workbench with the volar forearm facing upwards.

Before applying each formulation, a circular region, 24 mm in diameter, was defined on the treated area using a polyethylene adhesive template fixed to the arm. Three circular regions were defined by each template in order to simultaneously apply the film and the vitamin E containing cream. The third application site was left untreated to evaluate the vitamin E skin basal content.

The film formulation, cut into 24 mm diameter discs, was placed on the forearm skin surface and prewetted with a fixed amount of 0.5 mL water. After a few seconds of slight pressure, the film adhered to the skin and was left in place for 2 hours on the left forearm and for 4 hours on the right forearm. A commercial cream containing 3.6% vitamin E acetate was used as comparison. The vitamin E acetate containing semisolid formulation was applied at 15 mg/cm^2^ on a circular area of 24 mm diameter. The removal of *stratum corneum* was performed with 20 adhesive tape strips (Scotch 845, 3M, USA). Each strip was weighed before and after the application on the skin in order to evaluate the amount of *stratum corneum* removed.

For each experiment, the tape strips were pooled in test tubes in sets of 4 subsequent strips, defining five depths of *stratum corneum* penetration, from 1 superficial layer to 5 deep layers and then extracted with 5ml of methanol. After one hour, the solution was filtered through 0.45 *µ*m nylon filters and analyzed in HPLC.

### 2.10. Statistics

Data were evaluated by two-way analysis of variance (ANOVA) followed by Tukey's test (Kaleidagraph, Synergy Software, USA). The significance level applied was *P* < 0.05. The results of the experiments are expressed as the mean ± standard deviation.

## 3. Results and Discussion 

### 3.1. Film Characterization

Three types of films were manufactured according to Table 1. They differed for the amount of PVA and sodium alginate. Thickness, weight, and residual water content of the polymeric films are shown in Table 2. The films presented thickness lower than 80 *μ*m and had a weight per square centimeter close to 20 mg/cm^2^. The amount of vitamin E acetate in each film was about 3.5% by weight. They were flexible and resistant and could be easily handled, cut in the desired shape, and eventually deformed to adapt to the application site.

The films produced were shown to be completely solubilized after 24 hours of immersion in distilled water. This is very important in the case of wound treatment, in view of the amount of wound exudates that are produced and which solubilize the film, causing the release of the film active substances.

Table 2 shows the characteristics and the mechanical properties of the films. The mechanical properties required are flexibility and resistance to facilitate their use as potential wound-dressing materials. The strength and elasticity of the film were measured by the parameters: Ts_*b*_ and *ε*_*b*_. The *ε*_*b*_ of the film decreased with the increase of alginate content from F1 to F3. The stronger and less elastic formulation was F3, with 2 : 1 ratio of alginate and hyaluronate. Formulation F1, with alginate/hyaluronate ratio 1 : 1, showed a higher elongation at break and lower tensile strength. This behavior could be due to the higher water content, provided by the hygroscopic contribution of hyaluronate. The intermediate formulation F2 showed a tensile strength equal to F1 and a percentage of *ε*_*b*_ not significantly different from F3. The films containing a 1.5 : 1 alginate/hyaluronate ratio could be a compromise for the application to damaged skin, providing a relatively low water content, good resistance but, at the same time, flexibility.

According to the classification of Krochta and de Mulder-Johnston [29], the results indicate that the films produced in our study exhibit good elongation at break. In their study, they reported that polymeric films of low-density polyethylene and polypropylene films showed an inverse relationship between tensile strength and elongation at break. Similarly, in the present study, the formulation F3 presenting less elongation is characterized by greater resistance, even if not significantly different from other formulations.

A factor that must be controlled is the amount of plasticizers. The addition of the plasticizer reduces the intermolecular forces along the polymer chains, thus improving the flexibility thereof, decreasing, however, the stress at break [30]. These interactions are needed to prevent the films from becoming fragile and brittle, facilitating their handling and use although their use promotes changes in the polymer structure. In the films prepared, the increase in alginate content led to a lower *ε*_*b*_ and higher Ts_*b*_ values despite a constant content of the plasticizer sorbitol. However, this could be significantly related to reduction in residual water content. In fact, the water molecules are generally regarded as the most natural plasticizers of films based on hydrocolloids. Similar results on the effect of moisture in tension and elongation at break were previously described for various hydrophilic films [31, 32]. A synergistic plasticizing effect of water and glycerol has been observed in carboxymethylcellulose films with consequent modification of mechanical properties and polymer glass transition temperature [27].

### 3.2. Scanning Electronic Microscopy Analyses

**Figure 1:**
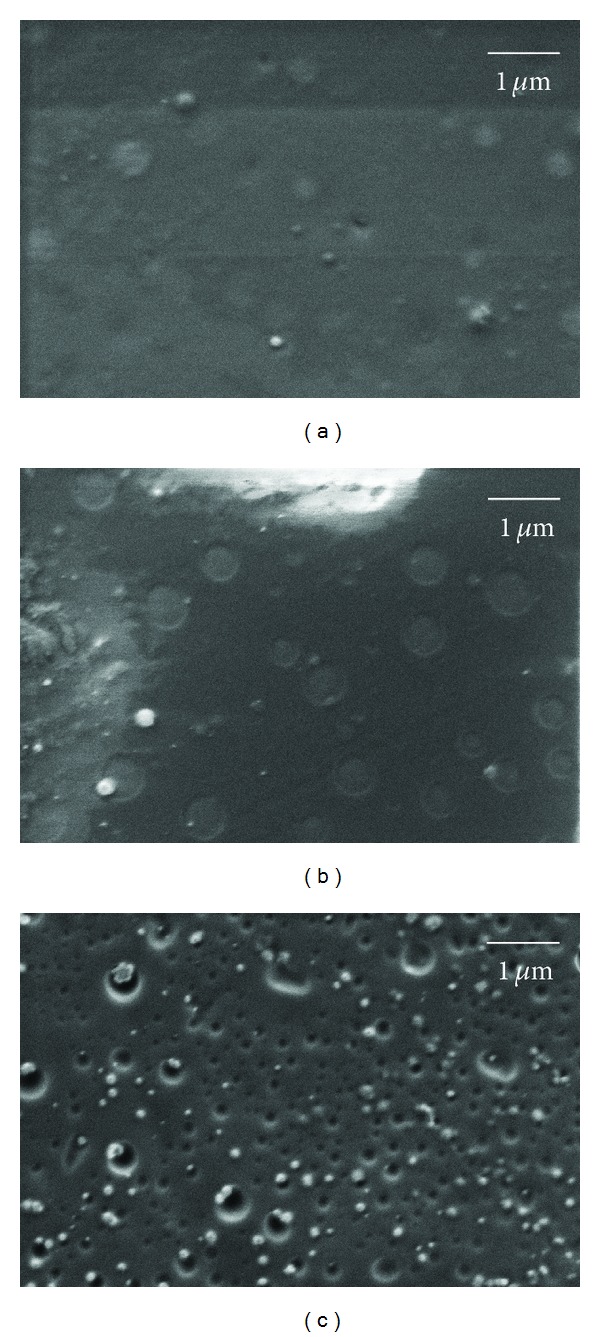
SEM images of polymeric films surface (a) polymeric film F1, (b) polymeric film F2, and (c) polymeric film F3. Magnification 50,000x. Film compositions are according to Table 1.

Scanning electron microscopy was used to visualize the film surface (Figures 1(a), 1(b), and 1(c)). Visual examination of the SEM pictures indicated that the films were essentially smooth, with a slightly rougher surface for formulation F3. The films analyzed by SEM showed no changes, heterogeneity, or component segregation detectable on the surface or cross-section. The film F3 presented dark pits, suggesting the presence of holes that arose during the drying process, possibly due to water separation, according to Dong and colleagues [22].

### 3.3. Atomic Force Microscopy (AFM)

**Figure 2:**
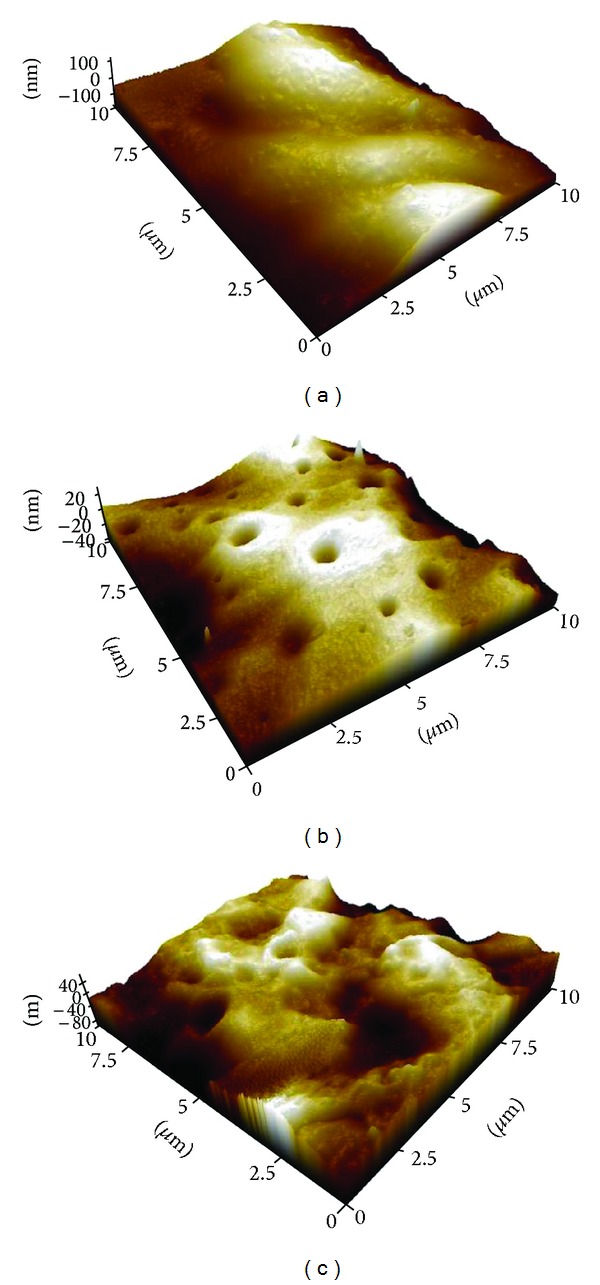
AFM images of polymeric films surface: (a) polymeric film F1 (b) polymeric film F2, (c) polymeric film F3. Film compositions are according to Table 1.

Using AFM, it was possible to visualize the details of the film surfaces (Figures 2(a), 2(b), and 2(c)). Film F1 presented in Figure 2(a) shows a smooth surface, while films F2 and F3 are characterized by a rougher surface with more frequent pits and depressions and of smaller dimensions.

The drying of the film resulted in pores, which were confirmed by SEM and AFM imaging. These pores increase the surface area, which could enhance the immediate release of the substance from the polymer surface [28]. Moreover, the micrometric roughness of the surface of the films can possibly allow them to offer a larger contact area to the healing tissue, which leads to a more efficient absorption of exudates, therefore enabling the promotion of a more efficient repair of the burn wound [33, 34].

### 3.4. *In Vitro* Vitamin E Release Studies

A high elasticity and strength, together with a considerable thickness and a sufficient drug content per square centimeter, led to the selection of the film formulation F2 for Franz cells and *tape stripping* experiments.

The study of the release profile provides essential information regarding the structure and the molecular behavior of the formulation, evidencing possible interactions between the drug and the polymer and their influence on the rate and mechanism of the drug release.

**Figure 3:**
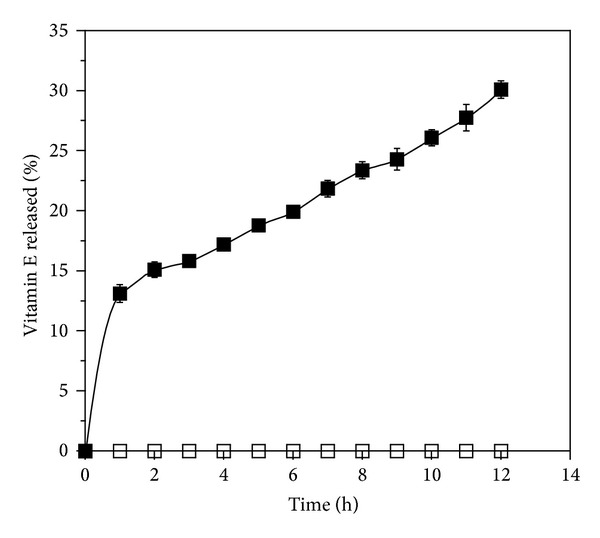
Vitamin E release from polymeric film F2 (■) and cream (□) in phosphate buffer pH = 7.2 containing 10% ethanol and 0.5% polysorbate 80. Bars represent standard derivations of means of five determinations (mean and SD, *n* = 5).

The release of vitamin E acetate from the polymeric film was 30.1% of the total after 12 h. The patterns of release of vitamin E acetate from polymeric films are shown in Figure 3. During the first hour of release, an immediate release of the vitamin occurred (around 13%) and after which the vitamin release rate remained fairly constant (1.55% per hour).

The amount of vitamin E acetate released after 12 hours was much higher for the polymeric film than the release measured in the case of the vitamin E acetate containing cream used for comparison. The amount of vitamin E acetate diffused from the cream was lower than 1% of the total amount applied.

The cream formulation showed a very strong affinity for the vitamin and hindering a partition with the water acceptor phase. This observation can be related to the viscosity of the formulation and to the low water solubility of the drug in the water media. In fact, discussing the smaller release of vitamin E acetate from O/W emulsions, some authors attributed this fact to the formation of a lamellar layer, formed by parallel surfactant bilayers separated by layers of aqueous solvent and forming a mono- or bidimensional network [35, 36]. This is responsible for the viscosity of these formulations and is eventually able to entrap the vitamin, due to the presence of multiple hydrophilic layers, in which the vitamin is poorly soluble, surrounding the oily phase in which vitamin E is dissolved.

Hydrophilic polymer films, like the formulation tested, provide a less important interaction with vitamin E acetate dispersed in them and a rapid water uptake, facilitating the release in the aqueous environment. A rapid water uptake with consequent immediate release, followed by the formation of a gel characterized by slower release, could explain the release pattern evidenced. The immediate release of the vitamin, shown at the beginning of the experiment, may has been prompted by vitamin present on the film surface. Subsequently, a gel layer is formed as a consequence of water uptake of the film and hydration of the hydrophilic polymers contained in it, that is, alginate and especially hyaluronic acid. This behavior in vitamin release is also consistent with the experimental setup: the donor compartment consists of a relatively small volume of liquid, which is rapidly absorbed by the hydrophilic film. This is mimicking the exudate absorption that would occur in a real burn wound.

### 3.5. Tape Stripping

The *in vivo* performance of the film was evaluated using the tape stripping technique to measure the accumulation of vitamin E acetate in the *stratum corneum* of intact skin after the film application. Tape stripping has become an investigative technique used in both research of topical bioavailability and bioequivalence, as proposed by FDA in 1998 [37]. Even if interaction with intact skin is not going to provide an evaluation of the efficacy of the formulation on burn wounds, it constitutes a good approach to compare the performance of the polysaccharide film against a traditional semisolid topical formulation.

The extraction of vitamin E acetate from the tape with *stratum corneum* resulted in specific and efficient recovery because no interference from skin components was present and the recovery was 101.65 ± 2.93%.

**Figure 4:**
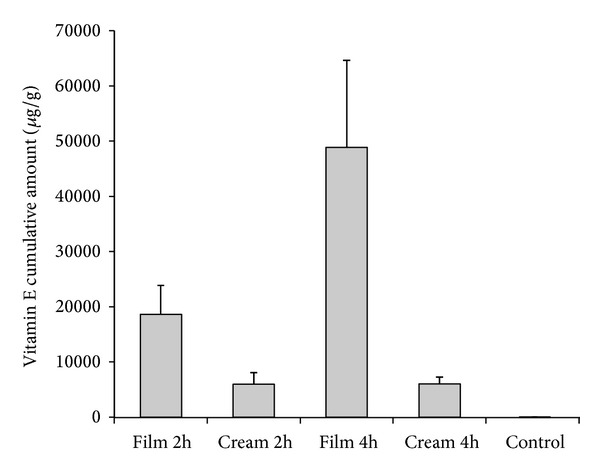
Cumulative vitamin E found per gram of skin from tape strips for different formulations. Untreated skin has been used as control (mean and SD, *n* = 5).

For each experiment, the tape strips were pooled in five sets numbered from 1 to 5 with pool 1 corresponding to the more superficial layers and pool 5 to the more deep layers of *stratum corneum*. The cumulative amount of vitamin E acetate recovered in the *stratum corneum* was also calculated (strips 1–20). The results obtained are reported in Figure 4. The comparison of the total recovery of vitamin E acetate in the *stratum corneum* after the application of the polymeric film or of the cream showed that the first was able to accumulate higher amounts of vitamin E acetate in the *stratum corneum* (see Figure 4). This was confirmed for both application times, that is, 2 and 4 hours. Furthermore, while the vitamin E acetate total recovery is the same for the cream between 2 and 4 hours, a much higher accumulation was registered for the film after 4 hours. This result indicates different release patterns.

**Figure 5:**
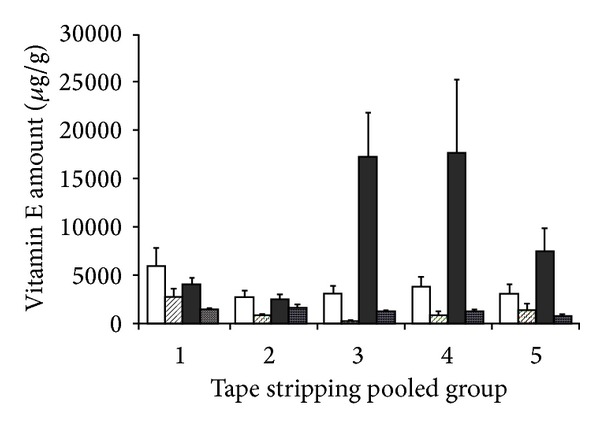
Amount of vitamin E from tape strips in pooled groups (mean and SD, *n* = 5) from polymeric film, 2 hours (white bar), polymeric film, 4 hours (black bar), cream, 2 hours (dashed bar), and cream, 4 hours (dotted bar).

From Figure 5, it is possible to appreciate that the vitamin E acetate contained in the films was able to permeate to the deeper layers of *stratum corneum* and in greater quantity than vitamin E acetate applied with the cream. It was also observed that the films that remained for four hours on the forearm of the volunteers showed a greater amount of total vitamin E acetate accumulated in those layers. The vitamin released by the film after 4 hours was able to reach the deepest layers of the *stratum corneum* in a significant amount and probably efficiently attain the viable skin layers. Vitamin E acetate content from the untreated skin was found to be negligible compared to the vitamin permeating from the formulations.

It is known that topical drugs are distributed first in the *stratum corneum* and from there are able to reach the dermis and epidermis. Therefore, the concentration of drug presence in the *stratum corneum* directly determines the concentration that diffuses to the other layers. So, skin bioavailability can be estimated by measuring the amount of drug present in the *stratum corneum* by the tape stripping technique [38, 39].

As shown in Figure 5, the amount of vitamin E acetate accumulated in different layers of the *stratum corneum* after application of the films was higher in the more internal layers and lower in the superficial portions of the *stratum corneum*, both after 2 and 4 hours of application. This evidence is consistent with some other studies in which it has been evidenced that the highest *α*-tocopherol levels were found in the lower *stratum corneum*, whereas the lowest levels were present in the upper layers [19].

Wound healing is a complicated process that requires several steps, encompassing the regenerative process initiated by the viable cells of the skin. Therefore, wounds such as burns heal from the inside out, so the amount of vitamin E that permeates more deeply in the skin aids in the healing process, acting in the deeper layers of the affected area. In particular, antioxidants, such as vitamin E, have been shown to play a significant role in wound healing and anti-inflammatory action. Preparations that increase free radical scavengers in situ can offer protection from cellular damage and facilitate the process of tissue repair [40].

Faster permeation through excised animal skin has been evidenced for polymeric transdermal patches developed by Padula and colleagues [5]. Those patches, called Patch-non-Patch, are applied by wetting the skin and favoring film hydration and adhesion. The higher permeation rates evidenced for a number of substances, compared to semisolid formulations, have also been attributed to the contribution of water to the increase in the constant diffusion of substances in the film and in the *stratum corneum* hydrated layers. A similar situation could be hypothesized here to explain the much higher availability in the *stratum corneum* of the vitamin E acetate through the polymeric film formulation. The hydrophilic components of the film provide adhesion in the presence of an aqueous medium as exudates and the colloidal gel formed by the hydration of the polymer chains allow a better permeation of the poorly soluble and hydrophobic vitamin E [39].

## 4. Conclusion 

Bioadhesive polymeric films loaded with vitamin E acetate and *Aloe vera* were produced and tested on the intact skin of healthy volunteers. The films showed mechanical resistance and flexibility suitable for application in burn wounds. The release profile obtained from the film showed a biphasic controlled release of vitamin E acetate for more than 12 hours. The *tape stripping* on intact skin showed that the polymeric film formulation facilitates a deeper accumulation of the vitamin E acetate in the *stratum corneum* when compared to a traditional semisolid formulation. The polymer film formulation containing hyaluronate and alginate appears to be a promising approach for the application of substances able to reduce damage and facilitate the healing process, like *Aloe vera* extracts and the antioxidant vitamin E acetate.

## Figures and Tables

**Table 1:** Percentage composition (% w/w) of vitamin E acetate and *Aloe vera* loaded polymeric films.

Components	F1	F2	F3
PVA (83,400 Da)	35.00	30.25	25.50
Hyaluronate	9.50	9.50	9.50
Sorbitol	27.90	27.90	27.90
PEO 12 NF (1000 kDa)	13.50	13.50	13.50
Sodium alginate	9.50	14.25	19.00
Vitamin E acetate	3.60	3.60	3.60
*Aloe vera *	1.00	1.00	1.00

**Table 2:** Characterization and mechanical properties of vitamin E and *Aloe vera* loaded polymeric films (*n* = 6).

	F1	F2	F3
Thickness (*μ*m)	58.00 ± 0.45^a^	74.00 ± 0.55	74.00 ± 0.54
Weight (mg/cm^2^)	20.70 ± 0.05	20.54 ± 0.08	20.66 ± 0.07
% Vitamin E (w/w)	3.54 ± 0.10	3.55 ± 0.10	3.55 ± 0.10
Water content (%)	5.36 ± 0.04^a^	2.95 ± 0.28^b^	1.48 ± 0.51
*ε*_*b*_ (%)	141.83 ± 12.43	132.78 ± 3.81	128.31 ± 2.62
Ts_*b*_ (N/mm^2^)	4.14 ± 1.24	4.13 ± 1.36	4.74 ± 1.41

^a^Mean and standard deviation, *n* > 6: significantly different when compared to F2 and F3 (*P* < 0.05).

^b^Mean and standard deviation, *n* > 6: significantly different when compared to F1 and F3 (*P* < 0.05).

## References

[1] Boateng JS, Matthews KH, Stevens HNE, Eccleston GM (2008). Wound healing dressings and drug delivery systems: a review. *Journal of Pharmaceutical Sciences*.

[2] Wolf SE, Sterling JP, Hunt JL, Arnoldo BD (2011). The year in burns 2010. *Burns*.

[3] Drago H, Marín GH, Sturla F (2010). The next generation of burns treatment: intelligent films and matrix, controlled enzymatic debridement, and adult stem cells. *Transplantation Proceedings*.

[4] Kirker KR, Luo Y, Nielson JH, Shelby J, Prestwich GD (2002). Glycosaminoglycan hydrogel films as bio-interactive dressings for wound healing. *Biomaterials*.

[5] Padula C, Colombo G, Nicoli S, Catellani PL, Massimo G, Santi P (2003). Bioadhesive film for the transdermal delivery of lidocaine: in vitro and in vivo behavior. *Journal of Controlled Release*.

[6] Sievens-Figueroa L, Bhakay A, Jerez-Rozo JI (2012). Preparation and characterization of hydroxypropyl methyl cellulose films containing stable BCS Class II drug nanoparticles for pharmaceutical applications. *International Journal of Pharmaceutics*.

[7] Andrew B, Hari GG, Hales CA (2004). Ch 18: Hyaluronan and scarring. *Chemistry and Biology of Hyaluronan*.

[8] Gong J, Chen M, Zheng Y, Wang S, Wang Y (2012). Polymeric micelles drug delivery system in oncology. *Journal of Controlled Release*.

[9] Davidson JM, Nanney LB, Broadley KN, Whitsett JS, Aquino AM (1991). Hyaluronate derivatives and their application to wound healing: preliminary observations. *Clinical Materials*.

[10] Goldberg SR, Diegelmann RF (2010). Wound Healing Primer. *Surgical Clinics of North America*.

[11] Jurjus A, Atiyeh BS, Abdallah IM (2007). Pharmacological modulation of wound healing in experimental burns. *Burns*.

[12] Dantas MDM, Cavalcante DRR, Araújo FEN (2011). Improvement of dermal burn healing by combining sodium alginate/chitosan- based films and low level laser therapy. *Journal of Photochemistry and Photobiology B*.

[13] Lademann J, Schanzer S, Richter H (2008). Formation of a protection film on the human skin by microparticles. *Laser Physics Letters*.

[14] Reynolds T, Dweck AC (1999). *Aloe vera* leaf gel: a review update. *Journal of Ethnopharmacology*.

[15] Choi S, Chung M-H (2003). A review on the relationship between *Aloe vera* components and their biologic effects. *Seminars in Integrative Medicine*.

[16] Cuttle L, Kempf M, Kravchuk O (2008). The efficacy of *Aloe vera*, tea tree oil and saliva as first aid treatment for partial thickness burn injuries. *Burns*.

[17] Maenthaisong R, Chaiyakunapruk N, Niruntraporn S, Kongkaew C (2007). The efficacy of *Aloe vera* used for burn wound healing: a systematic review. *Burns*.

[18] Zampieri N, Zuin V, Burro R, Ottolenghi A, Camoglio FS (2010). A prospective study in children: pre- and post-surgery use of vitamin E in surgical incisions. *Journal of Plastic, Reconstructive and Aesthetic Surgery*.

[19] Thiele JJ, Ekanayake-Mudiyanselage S (2007). Vitamin E in human skin: organ-specific physiology and considerations for its use in dermatology. *Molecular Aspects of Medicine*.

[20] Endre AB, Hari GG, Hales CA (2004). Ch 20: Viscoelastic properties of hyaluronan and its therapeutic use. *Chemistry and Biology of Hyaluronan*.

[21] Burd DAR, Greco RM, Regauer S, Longaker MT, Siebert JW, Garg HG (1991). Hyaluronan and wound healing: a new perspective. *British Journal of Plastic Surgery*.

[22] Dong Z, Wang Q, Du Y (2006). Alginate/gelatin blend films and their properties for drug controlled release. *Journal of Membrane Science*.

[23] Cencetti C, Bellini D, Pavesio A, Senigaglia D, Passariello C, Virga A (2012). Preparation and characterization of antimicrobial wound dressings based on silver, gellan, PVA and borax. *Carbohydrate Polymers*.

[24] Aramwit P, Siritienthong T, Srichana T, Ratanavaraporn J (2013). Accelerated healing of full-thickness wounds by genipin-crosslinked silk sericin/PVA scaffolds. *Cells Tissues Organs*.

[25] Peh KK, Khan TT, Ch’ng HS (2000). Mechanical, bioadhesive strength and biological evaluations of chitosan films for wound dressing. *Journal of Pharmacy & Pharmaceutical Sciences*.

[26] Ampollini L, Sonvico F, Barocelli E (2010). Intrapleural polymeric films containing cisplatin for malignant pleural mesothelioma in a rat tumour model: a preliminary study. *European Journal of Cardio-Thoracic Surgery*.

[27] Boateng JS, Stevens HNE, Eccleston GM, Auffret AD, Humphrey MJ, Matthews KH (2009). Development and mechanical characterization of solvent-cast polymeric films as potential drug delivery systems to mucosal surfaces. *Drug Development and Industrial Pharmacy*.

[28] Galdeano MC, Mali S, Grossmann MVE, Yamashita F, García MA (2009). Effects of plasticizers on the properties of oat starch films. *Materials Science and Engineering C*.

[29] Krochta JM, de Mulder-Johnston C (1997). Edible and biodegradable polymer films: challenges and opportunities. *Food Technology*.

[30] Guilbert S, Mathlouthi EM (1986). Ch 19: Technology and application of edible protective film. *Food Packaging and Preservation*.

[31] Cuq B, Gontard N, Cuq J-L, Guilbert S (1997). Selected functional properties of fish myofibrillar protein-based films as affected by hydrophilic plasticizers. *Journal of Agricultural and Food Chemistry*.

[32] Irissin-Mangata J, Bauduin G, Boutevin B, Gontard N (2001). New plasticizers for wheat gluten films. *European Polymer Journal*.

[33] Jimenez-Castellanos MR, Zia H, Rhodes CT (1993). Mucoadhesive drug delivery systems. *Drug Development and Industrial Pharmacy*.

[34] Shi C, Zhu Y, Ran X, Wang M, Su Y, Cheng T (2006). Therapeutic potential of chitosan and its derivatives in regenerative medicine. *Journal of Surgical Research*.

[35] Förster T, Jackwerth B, Pittermann W, von Rybinski W, Schmitt M (1997). Properties of emulsions. *Cosmetic Toiletries*.

[36] Csóka I, Csányi E, Zapantis G, Nagy E, Fehér-Kiss A, Horváth G (2005). In vitro and in vivo percutaneous absorption of topical dosage forms: case studies. *International Journal of Pharmaceutics*.

[37] Jacobi U, Weigmann H-J, Ulrich J, Sterry W, Lademann J (2005). Estimation of the relative stratum corneum amount removed by tape stripping. *Skin Research and Technology*.

[38] Padula C, Fulgoni A, Santi P (2010). In vivo stratum corneum distribution of lidocaine, assessed by tape stripping, from a new bioadhesive film. *Skin Research and Technology*.

[39] Padula C, Chiapponi C, Dibari MT (2010). Single layer transdermal film containing lidocaine: water and lidocaine mobility determined using neutron scattering. *Journal of Pharmaceutical Sciences*.

[40] Shukla A, Rasik AM, Patnaik GK (1997). Depletion of reduced glutathione, ascorbic acid, vitamin E and antioxidant defence enzymes in a healing cutaneous wound. *Free Radical Research*.

